# Supplementary material for: Unprecedented Fe delivery from the Congo River margin to the South Atlantic Gyre
Source: Nat Commun. 2020 Jan 28;11:556. doi: 10.1038/s41467-019-14255-2 (PMC6987306; doi:10.1038/s41467-019-14255-2)
Supplement: Supplementary file 1 — Supplementary Information [file 41467_2019_14255_MOESM1_ESM.pdf]

Supplementary Information for:

Unprecedented Fe delivery from the Congo River margin to the South Atlantic Gyre

by Vieira et al.

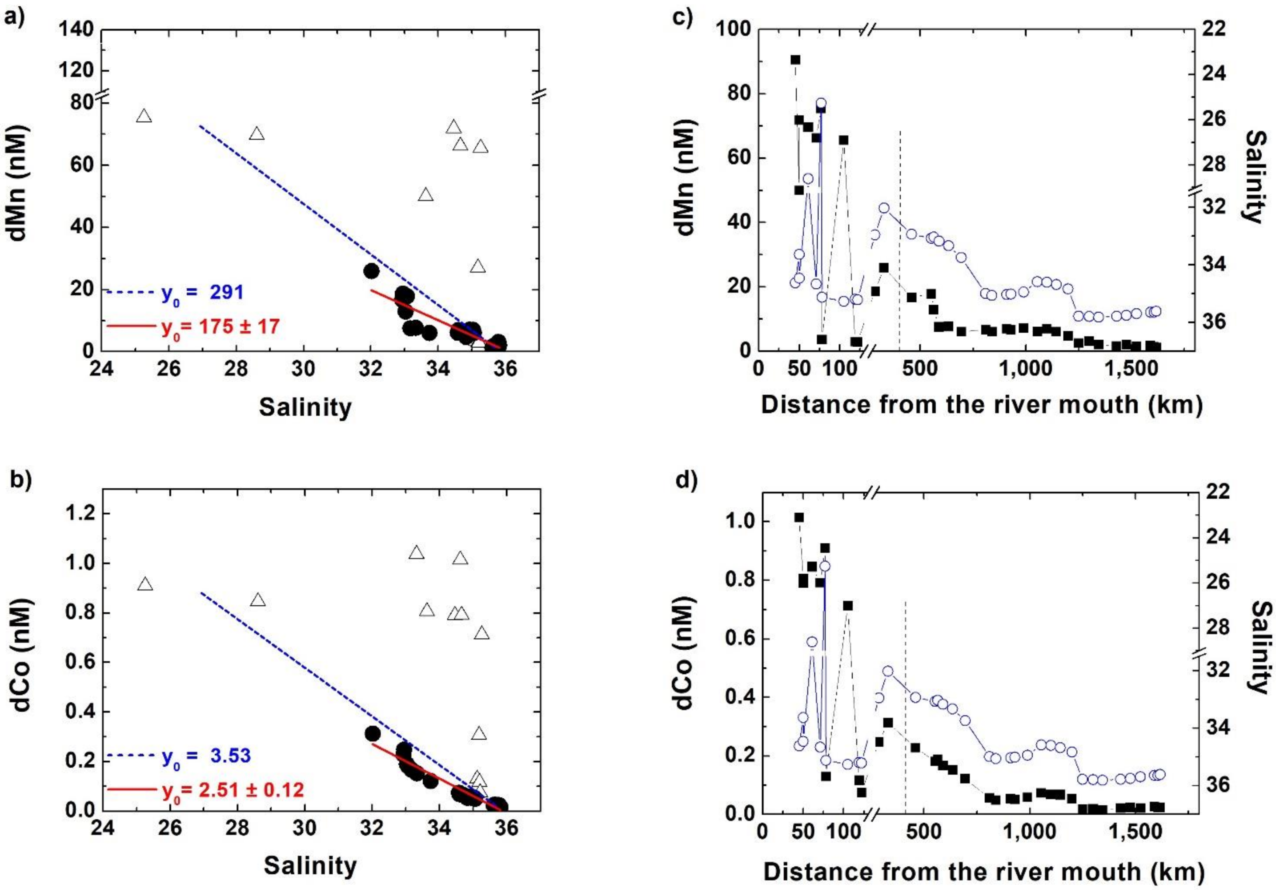

Supplementary Figure 1: Mixing diagram between river and open ocean waters from the Congo-shelf-zone to the end of the 3°S transect (st.1202-1247) for dMn (a) and dCo (b) concentrations. Open triangles represent the samples collected in the Congo-shelf-zones and circles represent the samples in the off-shelf transect. Dashed blue lines represent conservative mixing between freshwater and seawater, which connect two points that represent the lowest salinity (average of the samples with salinity < 29) and highest salinity (average of the samples with salinity > 36) measured during our whole study. Solid red line represents the linear regression for samples in the off-shelf transect (for TE); their intercept values (y_0) area notated. (c) and (d): dMn and dCo concentrations (solid black squares), and inverse salinity distributions (open blue circles) in surface waters from the Congo-shelf-zone to the end of the 3°S transect (st.1202-1247), respectively. Dashed vertical lines in (c) and (d) represent the beginning of the off-shelf transect at 3°S. Data provided in Source Data File.

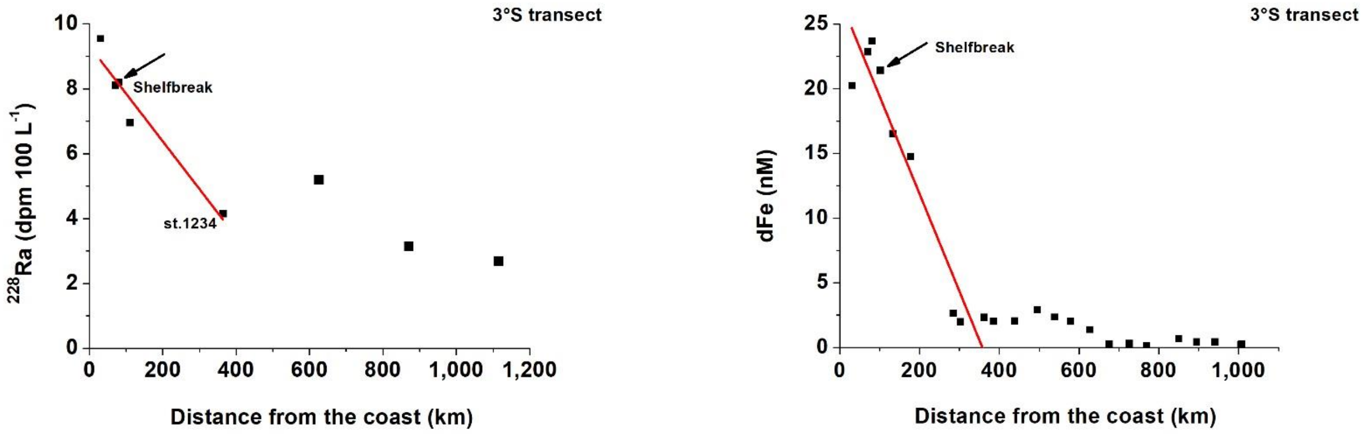

Supplementary Figure 2: ^{228}Ra and dFe distributions in surface waters of the 3°S transect. Red line represents the linear fit in the inner 360 km of the 3°S transect, and arrows represent the location of the shelfbreak. Data provided in Source Data File.

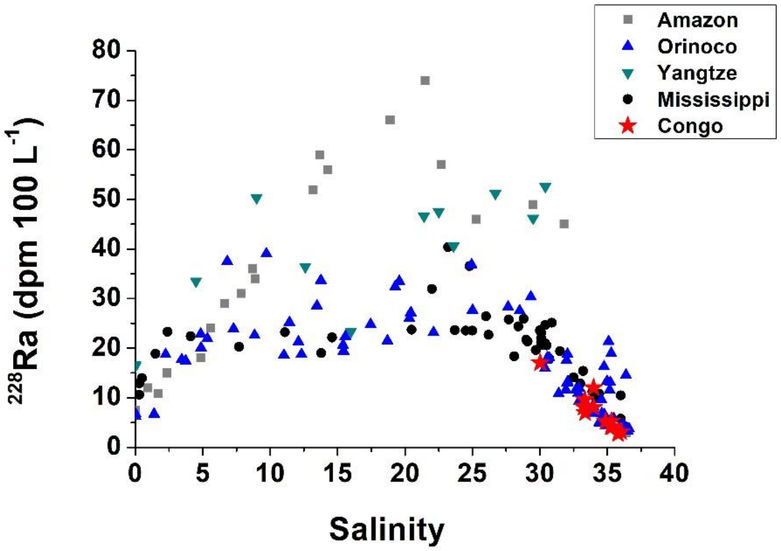

Supplementary Figure 3: Concentrations of ^{228}Ra across the estuarine salinity gradient in the Amazon[1], Orinoco[2], Yangtze[3], Mississippi[4] estuaries and this study. River endmembers range between 3.2 to 16.3 dpm 100 L^{-1}, with non-conservative input at mid-salinity.

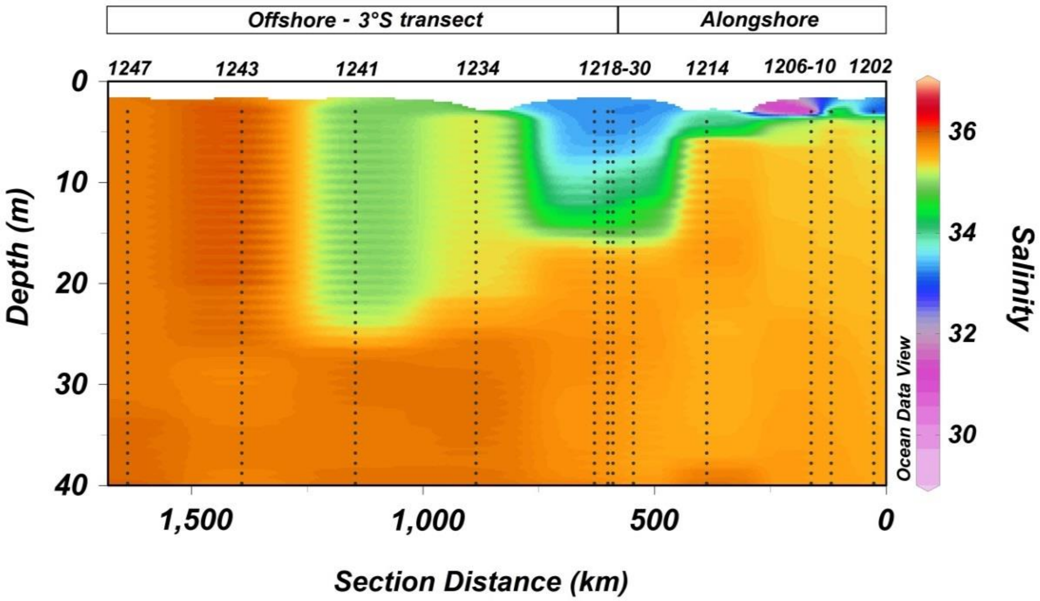

Supplementary Figure 4: Salinity profiles within the Congo River plume. Numbers on the top represent the stations sampled during this study (see Fig. 1 in the main text).

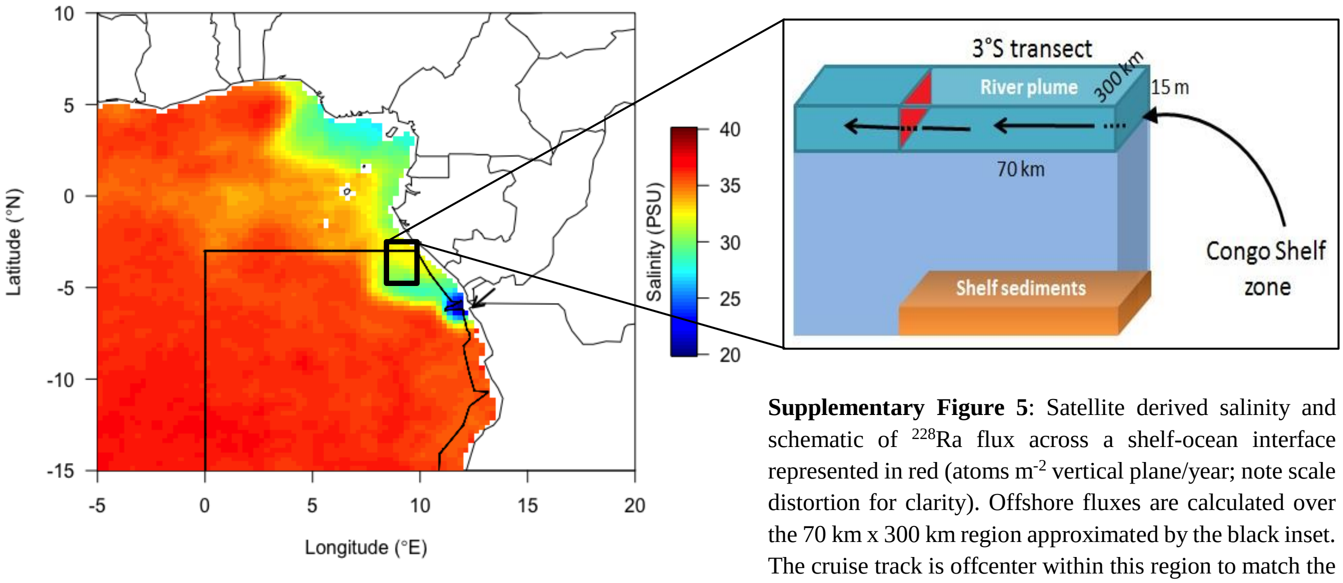

Supplementary Figure 5: Satellite derived salinity and schematic of ^{228}Ra flux across a shelf-ocean interface represented in red (atoms m^{-2} vertical plane/year; note scale distortion for clarity). Offshore fluxes are calculated over the 70 km x 300 km region approximated by the black inset. The cruise track is offcenter within this region to match the observed plume distribution.

Supplementary Table 1: Trace element concentrations measured in the Congo River at 6°027 S, 12°603 E.						
Collection date	dFe (nM)	dMn (nM)	dCo (nM)	TdFe (nM)	TdMn (nM)	TdCo (nM)
04.05.2017	10,827 ± 416	138 ± 4.2	2.4 ± 0.0	17,016 ± 877	138 ± 4.21	2.4 ± 0.0
22.07.2017	4,638 ± 219	76 ± 4.1	1.2 ± 0.2	19,975 ± 1290	250 ± 12	3.6 ± 0.1
08.10.2017	6,689 ± 771	97 ± 8.4	1.5 ± 0.4	11,046 ± 266	266 ± 17	3.3 ± 0.1

Supplementary Table 2: Values for TE analyses for SAFe S, D2 and CASS6 Certificate Reference Material (CRM)

	TEs	Consensus value (nM)	Reported value (nM)
SAFe S [a]	dMn	0.790 ± 0.060	0.860 ± 0.099 (n = 2)
	dFe	0.093 ± 0.008	0.106 ± 0.013 (n = 2)
	dCo	0.005 ± 0.001	0.004 ± 0.002 (n = 2)
CASS6 [b]	dMn	40.51 ± 2.23	42.8 ± 0.76 (n = 3)
	dFe	27.97 ± 2.19	26.79 ± 2.75 (n = 3)
SAFe D2 [a]	dCo	0.046 ± 0.003	0.054 ± 0.004 (n = 2)

[a] Bruland K.W., 2009. GEOTRACES and SAFe Intercalibrations, Consensus Values for the GEOTRACES 2008 and SAFe Reference Samples. In: http://es.ucsc.edu/~kbruland/GeotracesSaFe/kwbGeotracesSaFe.html (accessed 12 December 2019). SAFe S and D2 were determined via pre-concentration follow by ICPMS analysis.

[b] In: https://www.nrc-cnrc.gc.ca/eng/solutions/advisory/crm/certificates/cass_6.html (accessed 12 December 2019). CASS6 were determined by isotope dilution alongside high TE samples (> 20 nM Fe)

Supplementary Table 3: Analytical Blanks (n = 30)

	TEs	Value (pM)
System Blank (SeaFAST & ICP-MS)	dMn	9 ± 4
	dFe	61 ± 24
	dCo	1.9 ± 1
Buffer Blank	dMn	42 ± 15
	dFe	102 ± 53
	dCo	2.6 ± 2

Supplementary Note 1

Throughout the SE Atlantic region, Congo River waters are confined to the upper layers, which are decoupled from bottom influence[5, 6]. Proceeding downstream within the Congo River estuary, the bathymetry drops abruptly to 100 m depth due to the presence of a deep canyon at the river mouth which exerts a strong influence on the hydrography of the plume and ensures its detachment from shelf sediments[7]. Seasonal variations in wind direction and intensity strongly affect the Congo River plume dispersion[8, 9], as well as the complex circulation within the SE Atlantic Ocean[10]. Congo River plume dynamics have not been thoroughly investigated and models concerning its off-shelf distribution are not entirely consistent[9, 11, 12, 13]. Some studies suggest that the typical orientation is west-north-west, due to a combination of the unique geomorphology of the Congo River estuary, ocean currents and wind patterns[6, 9, 14]. A more recent study however suggests that the near equatorial river discharge of the Congo River generates a β-plume and is characterized by a train of eddies propagating westward[13].

References

1. Key, R. M., Stallard, R. F., Moore, W. S. & Sarmiento, J. L. Distribution and Flux of ^{226}Ra and ^{228}Ra in the Amazon River Estuary. *J. Geophys. Res.* **90**, 6995–7004 (1985).

2. Moore, W. S. & Todd, J. F. Radium isotopes in the Orinoco Estuary and eastern Caribbean Sea. *J. Geophys. Res.* **98**, 2233–2244 (1993).

3. Elsinger, R. J. & Moore, W. S. ^{226}Ra and ^{228}Ra in the mixing zones of the Pee Dee River-Winyah Bay, Yangtze River and Delaware Bay Estuaries. *Estuar. Coast. Shelf Sci.* **18**, 601–613 (1984).

4. Krest, J. M., Moore, W. S. & Rama. ^{226}Ra and ^{228}Ra in the mixing zones of the Mississippi and Atchafalaya rivers: Indicators of groundwater input. *Mar. Chem.* **64,** 129–152 (1999).

5. Yankovsky, A. E. & Chapman, D. C. A simple theory for the fate of buoyant coastal discharges. *J. Phys. Oceanogr.* **27**, 1386–1401 (1997).

6. Hopkins, J. *et al.* Detection and variability of the Congo River plume fromsatellite derived sea

surface temperature, salinity, ocean colour and sea level. *Remote Sens. Environ.* **139**, 365–385 (2013).

7. Jansen, J. H. F., Giresse, P. & Moguedet, G. Structural and sedimentary geology of the Congo and Southern Gabon continental shelf; a seismic and acoustic reflection survey. *Netherlands J. Sea Res.* **17**, 364–384 (1984).

8. Signorini, S. R., Murtugudde, R. G., Mcclain, C. R., Christian, P. J. R. & Busalacchi, A. J. Biological and physical signatures in the tropical and subtropical Atlantic. *J. Geophys. Res.* **104**, 18367–18385 (1999).

9. Denamiel, C., Budgell, W. P. & Toumi, R. The Congo River plume: Impact of the forcing on the far-field and near-field dynamics. *J. Geophys. Res. Ocean.* **118**, 964–989 (2013).

10. Strammar, L. & Schott, F. The mean flow field of the tropical Atlantic Ocean. *Deep. Res.* **46**, 279–303 (1999).

11. Vic, C., Berger, H., Tréguier, A.-M. & Couvelard, X. Dynamics of an Equatorial River Plume: Theory and Numerical Experiments Applied to the Congo Plume Case. *J. Phys. Oceanogr*. **44**, 980–994 (2014).

12. Nof, D., Pichevin, T. & Sprintall, J. “Teddies” and the Origin of the Leeuwin Current. *J. Phys. Oceanogr*. **32**, 2571–2588 (2002).

13. Palma, E. D. & Matano, R. P. Journal of Geophysical Research : Oceans An idealized study of near equatorial river plumes. *J. Geophys. Res. Ocean.* **122**, 3599–3620 (2017).

14. Eisma, D. & van Bennekom, A. J. The Zaire River and estuary and the Zaire outflow in the Atlantic Ocean. *Netherlands J. Sea Res*. **12**, 255–272 (1978).
