# Supplementary material for: Unprecedented Fe delivery from the Congo River margin to the South Atlantic Gyre
Source: Nat Commun. 2020 Jan 28;11:556. doi: 10.1038/s41467-019-14255-2 (PMC6987306; doi:10.1038/s41467-019-14255-2)
Supplement: Supplementary file 3 — Reporting Summary [file 41467_2019_14255_MOESM3_ESM.pdf]

nature research

Corresponding author(s): Lúcia H. Vieira

Last updated by author(s): Dec 13, 2019

Reporting Summary

Nature Research wishes to improve the reproducibility of the work that we publish. This form provides structure for consistency and transparency in reporting. For further information on Nature Research policies, see Authors & Referees and the Editorial Policy Checklist.

Statistics

For all statistical analyses, confirm that the following items are present in the figure legend, table legend, main text, or Methods section.

n/a	Confirmed	
☐	☒	The exact sample size (*n*) for each experimental group/condition, given as a discrete number and unit of measurement
☐	☒	A statement on whether measurements were taken from distinct samples or whether the same sample was measured repeatedly
☒	☐	The statistical test(s) used AND whether they are one- or two-sided<br>*Only common tests should be described solely by name; describe more complex techniques in the Methods section.*
☒	☐	A description of all covariates tested
☒	☐	A description of any assumptions or corrections, such as tests of normality and adjustment for multiple comparisons
☐	☒	A full description of the statistical parameters including central tendency (e.g. means) or other basic estimates (e.g. regression coefficient) AND variation (e.g. standard deviation) or associated estimates of uncertainty (e.g. confidence intervals)
☒	☐	For null hypothesis testing, the test statistic (e.g. *F*, *t*, *r*) with confidence intervals, effect sizes, degrees of freedom and *P* value noted<br>*Give P values as exact values whenever suitable.*
☒	☐	For Bayesian analysis, information on the choice of priors and Markov chain Monte Carlo settings
☒	☐	For hierarchical and complex designs, identification of the appropriate level for tests and full reporting of outcomes
☒	☐	Estimates of effect sizes (e.g. Cohen's *d*, Pearson's *r*), indicating how they were calculated

Our web collection on statistics for biologists contains articles on many of the points above.

Software and code

Policy information about availability of computer code

Data collection	No computer code was used
Data analysis	NA

For manuscripts utilizing custom algorithms or software that are central to the research but not yet described in published literature, software must be made available to editors/reviewers. We strongly encourage code deposition in a community repository (e.g. GitHub). See the Nature Research guidelines for submitting code & software for further information.

Data

Policy information about availability of data

All manuscripts must include a data availability statement. This statement should provide the following information, where applicable:

- Accession codes, unique identifiers, or web links for publicly available datasets
- A list of figures that have associated raw data
- A description of any restrictions on data availability

All new data is available in the main text or the Supplementary Info. Source data for dTE and Ra concentrations are provided as a Source Data file. See also DOI:10.2312/cr_m121

Field-specific reporting

Please select the one below that is the best fit for your research. If you are not sure, read the appropriate sections before making your selection.

☐ Life sciences ☐ Behavioural & social sciences ☒ Ecological, evolutionary & environmental sciences

Ecological, evolutionary & environmental sciences study design

All studies must disclose on these points even when the disclosure is negative.

Study description	Radium-228 was used as a geochemical tracer to track the horizontal trace element (dFe, dCo, and dMn) dispersions of the Congo River plume and to quantify the input rates of these elements from the Congo River into the South Atlantic Gyre.
Research sample	Surface seawater samples for Ra isotope and dissolved trace element (dTE) analyses.
Sampling strategy	Surface seawater samples were collected onboard R/V Meteor during the GEOTRACES GA08 cruise in the Southern Atlantic. See DOI:10.2312/cr_m121
Data collection	MnO2-impregnated acrylic fibers were used to quantitatively extract Ra isotopes, whose activities were determined using a delayed coincidence counter (RaDeCC) and gamma spectrometer. Trace element samples were collected using a tow fish deployed alongside the ship, and elements were preconcentrated using an automated system (SeaFAST, ESI) with subsequent analysis using a high resolution inductively coupled plasma-mass spectrometer. L.H.V, M.J.H., and E.P.A. collected the samples. L.H.V, M.J.H., and S.K. measured the samples and analyzed the data.
Timing and spatial scale	November-December 2015 in the Southeast Atlantic Ocean.
Data exclusions	No data were excluded.
Reproducibility	GEOTRACES protocols were applied throughout sampling and analysis.
Randomization	NA
Blinding	NA

Did the study involve field work? ☒ Yes ☐ No

Field work, collection and transport

Field conditions	See DOI:10.2312/cr_m121
Location	Southeast Atlantic Ocean, between 3 and 8°S; 11 and 0°E
Access and import/export	See cruise report: DOI:10.2312/cr_m121
Disturbance	NA

Reporting for specific materials, systems and methods

We require information from authors about some types of materials, experimental systems and methods used in many studies. Here, indicate whether each material, system or method listed is relevant to your study. If you are not sure if a list item applies to your research, read the appropriate section before selecting a response.

Materials & experimental systems

n/a	Involved in the study	
☒	☐	Antibodies
☒	☐	Eukaryotic cell lines
☒	☐	Palaeontology
☒	☐	Animals and other organisms
☒	☐	Human research participants
☒	☐	Clinical data

Methods

n/a	Involved in the study	
☒	☐	ChIP-seq
☒	☐	Flow cytometry
☒	☐	MRI-based neuroimaging
